# New Radiobiological Principles for the CNS Arising from Space Radiation Research

**DOI:** 10.3390/life13061293

**Published:** 2023-05-31

**Authors:** Richard A. Britten, Charles L. Limoli

**Affiliations:** 1 EVMS Radiation Oncology, Eastern Virginia Medical School, Norfolk, VA 23507, USA; 2 Department Radiation Oncology, University of California-Irvine, Irvine, CA 92697, USA; climoli@uci.edu

**Keywords:** space radiation, radiobiological principles, cancer treatment

## Abstract

Traditionally, the brain has been regarded as a relatively insensitive late-reacting tissue, with radiologically detectable damage not being reported at doses < 60 Gy. When NASA proposed interplanetary exploration missions, it was required to conduct an intensive health and safety evaluation of cancer, cardiovascular, and cognitive risks associated with exposure to deep space radiation (SR). The SR dose that astronauts on a mission to Mars are predicted to receive is ~300 mGy. Even after correcting for the higher RBE of the SR particles, the biologically effective SR dose (<1 Gy) would still be 60-fold lower than the threshold dose for clinically detectable neurological damage. Unexpectedly, the NASA-funded research program has consistently reported that low (<250 mGy) doses of SR induce deficits in multiple cognitive functions. This review will discuss these findings and the radical paradigm shifts in radiobiological principles for the brain that were required in light of these findings. These included a shift from cell killing to loss of function models, an expansion of the critical brain regions for radiation-induced cognitive impediments, and the concept that the neuron may not be the sole critical target for neurocognitive impairment. The accrued information on how SR exposure impacts neurocognitive performance may provide new opportunities to reduce neurocognitive impairment in brain cancer patients.

## 1. Introduction

### 1.1. Status of CNS Radiobiology Research Start of the Millennium

The detonation of the atomic bombs at Hiroshima and Nagasaki, and the subsequent Cold War concerns about nuclear warfare, led to unprecedented advances in our knowledge of the effects of radiation on the human body. By the 1980s, the deleterious effects of radiation were firmly attributed to radiation-induced DNA damage leading to cell death, and radiation-induced cell-killing models dominated virtually all aspects of radiobiology. Acute and late effects in both malignant and normal tissues were adequately explained by models using α/β cell survival parameters, which in turn led to major advances in normal tissue sparing by dose fractionation, largely due to the concept of Biologically Effective Dose (BED).

The central tenet underlying most radiobiological models was that radiation-induced cell killing was most enhanced in rapidly proliferating cells. This opinion stemmed from empirical studies on normal tissue sequelae after radiation exposure. In the aftermath of the atomic bomb detonations, the dose dependency of the normal tissue damage induced was characterized and designated as Acute Radiation Syndrome. The rapidly proliferating tissues of the hematological/immune system were damaged by low (<6 Gy) radiation doses (hematopoietic syndrome), whereas the more slowly proliferating tissue of the gut was not impacted until 8–10 Gy had been received (gastrointestinal syndrome). The fact that the cerebrovascular (neurovascular) syndrome is not induced unless exposure exceeds 30 Gy led to the concept that the brain was a relatively insensitive organ compared to the gastrointestinal and hematopoietic systems. The status of the CNS as a radioresistant organ was further enhanced by the fact that no radionecrosis, demyelination, or overt histological changes were detected until high radiation doses (>60 Gy) were delivered to the brain. Furthermore, in 1968, Casserett’s classification listed mixed post-mitotic cells (e.g., neurons) as having the lowest level of radiosensitivity of all tissue types [1]. Based upon this premise, it was logical to assume that the brain regions where neurogenesis occurs (the hippocampus and olfactory lobe) would be most impacted by radiation exposure.

### 1.2. Radiation Treatment for Brain Cancers

In light of the well-documented role that the hippocampus plays in multiple cognitive processes, specifically in the formation of new memories about experienced events (episodic or autobiographical memory) [2,3], it was a reasonable assumption that the exposure of the hippocampus to high radiation doses would lead to cognitive impairments. Radiation therapy for brain cancers was delivered in a manner that spared the hippocampus from receiving high doses of radiation. Originally, whole-brain irradiation (WBI) with hippocampal dose sparing was the treatment of choice for brain metastasis [2], but WBI resulted in significant late neurological toxicities [3,4] in surviving patients. Partial brain irradiation by means of stereotactic radiosurgery (SRS) was thus introduced. Although WBI achieved superior control of distant brain recurrence, SRS after resection resulted in equivalent survival times and greater neurological preservation [5,6]. The use of SRS approaches to treat medulloblastoma patients similarly led to a greater preservation of intellectual performance than was observed with WBI [7].

Despite these refinements, neurocognitive impairment still remains an issue, and in fact, due to the increasing survival rates of pediatric brain cancer patients, the long-term sequela of cranial irradiation is of increasing concern. By the mid-1980s, both clinical [8,9,10] and laboratory [11,12] studies suggested that cognitive function was impacted at much lower doses (<1.5 Gy X-rays) than the threshold doses predicated using the prevalent (cell-killing-centric) models of radiation lethality. The early studies on the impact of space radiation (SR) exposures on the brain suggested that the conditioned taste aversion (CTA) response was impacted after exposure to 200 mGy X-rays and by a variety of SR ion species [13,14]. Concomitant with these studies, it was demonstrated that low (100 mGy) SR doses altered dopamine-mediated neurotransmission in the striatum [15].

At the start of the 1980s, when neurocognitive testing was conducted, such assessments primarily focused on working memory and verbal skills. However, over the last 40 years, neurocognitive sparing approaches have increasingly focused on preserving cognitive functions that impact the quality of life of the patients, e.g., intellect, emotion, and executive functions. The advent of more readily available proton therapy (PRT) facilities has raised the prospect of even greater neurocognitive sparing over that seen with SRS. The use of PRT has resulted in fewer decrements in cognitive performance than X-ray treatment (XRT), with better preservation of global IQ, perceptual reasoning, and working memory [16]. However, two cognitive functions were not preserved by either XRT or PRT: verbal reasoning and processing speed [16]. These processes are primarily regulated by the cortex of the brain as are many executive functions. Executive functions are a set of higher-order cognitive abilities involved in adapting and regulating behavior and involve processing information from different sensory modalities, memory retrieval, and updating emotion and reward evaluations and regulation of response systems [17]. As such, executive functions serve as a metacognitive, supervisory, or controlling system [18] and require integrated processing of information within and across neural networks. Protection of these cognitive processes can only be achieved by understanding these processes and how radiation exposure impacts them. In the last two decades, there have been tremendous advances in our understanding of how the brain works, and also due to NASA-funded research on how low radiation doses impact neurological function, particularly advanced executive functions.

### 1.3. NASA-Related Low-Dose Radiobiology Developments

When NASA proposed interplanetary exploration missions (originally the Constellation program in 2005, which eventually morphed into the current Artemis program) it was required to comply with the US Government’s Occupational Safety and Health Administration requirements and to conduct an intensive health and safety evaluation of the risks associated with the planned missions. This evaluation needed to include SR exposure. The deep-space radiation spectrum (Galactic Cosmic Radiation (GCR)), is composed of highly energetic, high-mass (Z ≤ 28) charged ions, which are very hard to shield against, and very little was known about the biological effects of being exposed to the mixture of particles. Current estimates suggest that astronauts will be exposed to ~30 mGy of SR during each year of a mission to Mars (assuming current shielding/construction configurations) [19]. Based upon the current spacecraft design specifications, the majority of both the physical and biologically effective SR dose is predicted to arise from Z < 15 particles [20]. The unique nature of the SR made it difficult to extrapolate from the existing radiobiological risk models; however, the predicted dose for astronauts on a mission to Mars would be ~300 mGy, a level well below that considered to be of concern to most people at the time, based upon the cell-killing (DSB) models for radiation effects.

## 2. Space Radiation Effects on the CNS

In the early 1990s, much of the CNS radiobiology research focused on hippocampal-dependent cognitive processes, given the important role that the hippocampus plays in regulating several cognitive processes (learning, memory, pattern separation, and cognitive flexibility). This led to extensive efforts to establish the impact of SR on hippocampal neurogenesis and performance in tasks regulated by the hippocampus. Spatial learning and memory were investigated using the Morris water [21,22,23], Barnes [24,25], and radial arm [26,27,28] mazes. Learning and memory were also investigated using the Novel Object Recognition (NOR) test [29,30,31,32,33,34].

Due to the widely accepted concept that CNS cell killing was the primary determinant of cognitive impairment, the initial studies on the impact that SR particles (e.g., 1 GeV/n ^56^Fe) had on the CNS-employed SR doses that were BED to the threshold doses (TD) for neurocognitive impairment following X-ray exposure. Typically, the X-ray TD was divided by the relative biological effectiveness (RBE) weighting factors for cell killing for the particular SR particle studied (e.g., 1 GeV/n ^56^Fe has an RBE of 3.3 for cell killing [35]). However, cognitive impairment continued to be reported as progressively lower SR doses than the predicted BED were used. The RBE values for cognitive impairment following SR (compared to X-ray exposure) were frequently greater than that for the RBE for cell killing (e.g., RBEcog ≥ 10 [13], whereas the RBE for cell killing is 3.3). If the widely accepted concept that CNS cell killing was the primary determinant of cognitive impairment, then the TD for the impairment of a specific cognitive process should be constant for all SR particles when the RBE weighting factor for cell killing for each particle is applied to generate a BED dose (BEDκ) (Equation (1))

TD_SR1_ × RBE_SR1_ = BED_κ_; TD_SR2_ × RBE_SR2_ = BEDκ; TD_SRn_ × RBE_SRn_ = BEDκ. (1)

A 2006 analysis (Britten, NASA grant application 2006) of the published data from the Rabin laboratory (which had studied the effect that four different SR particles had on four different cognitive and motor function end-points) revealed that in fact there was a 7.5- and 13-fold discrepancy in the BED for impairment of operant response and conditioned taste aversion, respectively, (Table 1) when data from four different SR ions were analyzed. These findings started to raise questions about whether inhibition of neurogenesis was the sole cause of SR-induced impairment of neurocognition.

Unfortunately, few studies have concomitantly assessed the impact of SR and X-rays on cognitive performance; thus, it is difficult to accurately calculate RBE for cognitive impairment. However, when such data are available, RBE for cognitive impairment is markedly greater than the RBE for cell killing (~500 for He-induced decrements in the elevated plus maze [38] and >200 for Fe-induced decrements in spatial memory [24]).

## 3. New Perspective on Space Radiation Effects on the CNS

### 3.1. Paradigm Shift 1: Radiation-Induced Cell Death Is Not the Sole Determinant of Neurocognitive Impairment

Although SR exposure impairs neurogenesis [21,27,39,40,41,42,43,44], in some instances reduced neurogenesis was associated with impaired behavior/cognitive performance [39,41,44] whereas in others there was no obvious relationship between these two endpoints [39,40]. Furthermore, in some cognitive tasks, decreased hippocampal neurogenesis may enhance performance by diminishing memory interference [45,46], and enhancing sparse encoding [47,48].

As survival rates increased and more advanced cognitive tests (e.g., Trail Making Task-A, Wechsler’s, Controlled Oral Word Association) of executive function performance were employed on brain cancer patients, there was an increased awareness that cortex-dependent executive functions, such as attention and processing speed were negatively impacted by radiation exposure. Similarly, the focus of NASA-funded studies on SR-induced neurocognitive impairment began to shift towards cortex-dependent executive functions that had more operational significance. Over the last decade, rodent models demonstrated that SR exposure impairs performance in many cognitive tasks (reviewed in [39,49,50,51,52]), including cognitive flexibility. Cognitive flexibility is a critical executive function that can be broadly defined as the ability to adapt behaviors in response to changes in the environment. It is thus concerning that SR exposure (≤250 mGy) negatively impacts various aspects of cognitive flexibility in rodents [22,30,32,33,39,53,54,55,56,57,58,59,60,61,62,63,64,65]. Several cognitive processes, such as attention, task switching, and inhibition are involved in cognitive flexibility, with numerous studies reporting that low-dose (≤250 mGy) SR exposure impacts attention [33,53,54,55,57,59,60,61,62,63,64,65].

Logistical constraints have limited detailed investigations on how rapidly SR-induced performance decrements are induced, but such studies suggest that there are SR-induced decrements in cognitive [66] and sensorimotor [67] performance within three days of exposure. Similarly, there has not been a systematic evaluation of how persistent SR-induced performance decrements are. In general, most investigators report that SR-induced cognitive decrements are persistent, and in some cases may worsen due to concomitant age-related performance decrements [68]. However, it should be noted that in most studies, irradiated individuals are not required to utilize the cognitive processes in the period between SR exposure and cognitive assessments. Given the marked improvements in cognitive performance in stroke victims as a result of rehabilitative approaches, similar approaches may help to ameliorate SR-induced cognitive performance decrements [69].

The impairment of executive function performance after exposure to low radiation doses (~750 mGy BED) may mean that even sophisticated conformal treatment planning may be insufficient to spare these functions. It is interesting to note that performance in two executive functions (verbal reasoning and processing speed) was not spared using intensity-modulated proton therapy [16]. Conceptually, these data suggest that classic cell-killing models are less applicable to the CNS, at least for cortex-dependent cognitive processes. The classic concept that radiation-induced cognitive impairment occurs due to the loss of neurons would thus appear to be no longer valid. Ultimately, cognitive performance (memory and learning) reflects the functionality of the neurons; thus, anything that interferes with the ability of neurons to encode, store, and retrieve memories will impact cognition. Therefore, alternate mechanisms could include (1) loss or reduced functionality of individual neurons; (2) loss or compromised coordination within neural networks in the brain that regulate specific tasks; or (3) a combination of both.

Loss of neuronal functionality can occur via multiple mechanisms that do not involve radiation-induced DNA damage, or at least damage produced directly from the incident photons or charged SR particles. For decades, research has identified that the irradiated brain perhaps never returns to basal or “normal” states, and earlier work clearly pinpointed secondary reactive processes, including oxidative stress and inflammation as contributory if not causal to long-term disruptions in CNS functionality. Cascades of oxidative and inflammatory factors were found to persist in the irradiated brain after more clinically relevant doses [70,71,72]. Cellular studies over the years have identified radiation-induced disruptions to mitochondrial function as a source of reactive oxygen species (ROS) able to perpetuate damage signatures across multiple cellular compartments (membrane, cytoplasmic, nuclear) and macromolecules (lipids, proteins, nucleic acids) [73,74]. Leaky electron transport releases variable yields of superoxide, a relatively unreactive moiety but one that can participate in reactions that produce more powerful intracellular oxidants such as peroxynitrite and hydrogen peroxide. Hydrogen peroxide can diffuse throughout the cell and oxidize labile iron to produce highly reactive hydroxyl radicals [75]. Radiation exposure disrupts oxidative phosphorylation to potentiate mitochondrial-derived ROS and elevate oxidative stress over protracted post-irradiation intervals [73,76,77,78]. Work from us and others has substantiated this idea considerably [79,80,81], highlighting the many ways in which normal tissues and cancer cells differentially process and remove organic hydroperoxides and other byproducts of oxidative injury. Stem cells of the CNS have been found to contain higher ROS levels than in more mature progeny, likely due to more efficient scavenging systems that help maintain functional reserves [82]. However, their minor contribution to CNS cellularity renders these small differences in ROS levels between unique stem cell populations in the neurogenic regions of the brain of little consequence to space radiobiology. Early cellular work from our group using neural stem cells exposed to relatively higher doses of SR ions (protons and iron) found a certain dose-dependent increase in ROS and reactive nitrogen species (RNS) over week–month post-exposure timeframes [74,83,84]. Similar work with very low-dose iron ion exposures confirmed the capability of SR particles to elicit a significant and prolonged increase in several ROS and RNS species. When these studies were replicated in mice, a compensatory response was found in the rodent brain that resulted in a significant increase in antioxidant capacity two weeks after exposure before returning to baseline two weeks later [85]. Whereas rodent and human studies have attempted to pinpoint oxidative, metabolic, and lipid biomarkers of spaceflight stressors [86,87], and despite mitochondrial stress being found as a strong candidate for mediating many critical aspects of the CNS SR response, definitive signatures of radiation injury and biomarkers able to meaningfully inform on cognitive dysfunction have remained elusive [88].

The physical conformation of neurons results in quite unique 3D spatial distributions of the soma and nucleus compared with many cell types. In general, neurons have a tree-like structure including the soma which contains the cell nucleus, numerous dendritic branches, and a single axon [89]. Spatially, the dendritic tree occupies a much larger volume than does the soma (nucleus) of the neuron and thus has a higher probability of being subjected to ionization events. Modeling studies suggest that dendrites and spines within the dendritic tree are exposed to around 2- to 20-fold more dose, respectively, than the soma when 100 mGy of Fe is delivered to the brain [90]. It is, thus, perhaps not surprising that exposure to low SR doses results in significant and persistent changes in dendritic structure [33,91]. Coincident with these structural changes are microscopic alterations in the dendritic spines [39,90] and axons [91]. The demyelination of the axons would compromise conduction velocity, which when considered with the changes in dendritic structure, suggests that SR exposure impairs neurotransmission at multiple levels.

The SR-induced changes in dendritic arborization and complexity are likely to have profound consequences. The dendritic tree contains the synaptic sites that communicate with adjacent neurons. Dendritic morphology and spine numbers are indicative of synaptic function and frequently correlate with behavioral outcomes [92,93,94,95,96]. Thin dendritic spines are involved in mnemonic memory [97,98,99] and their numbers reflect the dendritic reserve available to “learn” a memory. Mushroom spines are indicative of stable memory [97,100,101,102] and regulate postsynaptic cell excitability, synaptic plasticity, and thus, synaptic strength. The number of mushroom spines, length of the neck, and diameter of the mushroom head can be used to assess memory in patients with neurological disorders [103]. SR exposure results in a significant loss in dendritic spine density of all conformations [33].

Given these changes in dendritic morphology, it is perhaps not surprising that radiation exposure (both X-rays and SR) alters synaptic functionality [62,63,104,105,106,107,108] and produces long-term potentiation (LTP) decrements in hippocampal and PFC synapses [109,110,111,112,113], long-term depression (LTD) decrements in the prefrontal cortex (PFC) [54]), and alters both excitatory and inhibitory neurotransmission [106].

### 3.2. Paradigm Shift 2: Non-Neuronal Basis for Radiation-Induced Neurocognitive Impairment

Although it is evident that SR exposure alters neuronal functionality, it would be inaccurate to assume that the neuron is the target cell for radiation-induced neurocognitive impairment. Historically, neurons have been the principal cell type investigated since they execute and control almost all the brain functions such as recognition, memory, depression, anxiety, etc. However, the brain contains both neuronal and non-neuronal cells. Non-neuronal cells vastly outnumber neurons and primarily included pericytes, endothelia, glial cells, etc. Glial cells can be subclassified into three types: microglia, astrocytes, and oligodendrocytes. In adult brains, microglia and astrocytes closely interact with neurons to modulate neuronal excitability and play a crucial role in shaping and maintaining the optimal synaptic network (e.g., [114,115,116]).

Astrocytes reciprocally interact with neurons within the “tripartite synapse”, consisting of two neurons and one astrocyte. Thus, astrocytes have to be considered as potential targets as well, and glutamate transporter activity within astrocytes is indeed reduced after exposure to SR [117]. Astrocytes do not only act as “helper” cells by regulating energy supply to neurons [118,119,120], they play a key role in modulating the structure and function of both excitatory and inhibitory synapses via the release of transmitters (such as glutamate) that target both pre- and post-synaptic sites. Conversely, astrocytes are the target of neurotransmitters released from neurons, which results in the activation of signaling pathways in the astrocytes, which in turn modulate synaptic behavior. The involvement of oligodendrocytes in regulating myelination is well-documented, and the significant loss of myelinated axons after SR exposure [121] is indicative of oligodendrocyte function being impaired. A less-appreciated function of oligodendrocytes is to provide metabolic support to neurons, rapidly transferring energy metabolites such as pyruvate and lactate to neurons [122]. It seems unlikely that SR would only impact the myelination functions of the oligodendrocytes and not their important neuronal homeostatic functions.

Microglia serve three essential functions in the brain: (1) act as environmental sentinels; (2) conduct physiological housekeeping; and (3) protect the brain from pathogens or other insults. Microglia are always active, not only screening for neuronal damage, but also actively communicating with both neuronal and non-neuronal cells, e.g., microglia neuronal excitability through secreted cytokines and chemokines (including the complement C1, CD200-CD200 receptor, and CX3CR-CX3CL1 axis) [123,124,125].

SR exposure has been reported to result in persistent neuroinflammation involving significant increases in activated microglia [33,40,85,91]. Persistent neuroinflammation is likely to elicit signaling changes that will disrupt homeostatic synaptic plasticity by altering the balance between excitatory and inhibitory neurotransmission. Thus, an SR-induced neuroinflammatory response is likely to alter the functionality of multiple neural circuits and thus cognitive performance. Elimination of microglia following radiation exposure reduces/ameliorates the loss of neurocognitive function that would otherwise occur [126,127]. These studies highlight the importance of neuroinflammation in dictating the long-term radiation response of the brain.

As alluded to above, the neurogenic regions of the brain contain the proliferative cells of the brain and are composed of quiescent stem cells and their immediate progeny. Neural stem cells are typically viewed as more resistant to radiation, whereas their progenitor cell pools exhibit exquisite sensitivity to photon and SR particle radiation exposure [40,41,128,129]. Post-mortem isotopic (^14^C) analysis of human brains analyzed before and after atomic bomb testing determined that neurogenesis added ~700 new neurons to each hippocampus per day, corresponding to an annual turnover of 1.75% of the renewable neuronal pool [130]. Clearly, the SR-induced depletion of the neurogenic precursor pool does not provide a plausible explanation for the manifold changes in cognition reported after a variety of exposure scenarios, nor do SR-induced alterations to the neurogenic microenvironment that might be non-permissive for mature neuronal differentiation. In sum, although the negative impact of SR on the neurogenic regions of the brain is multifaceted, increased cell attrition or altered trophic fate may only account for a relatively minor fraction of the global and network levels changes in neurotransmission able to afford functional cognitive change.

Other work provides hints that the approaches geared toward targeting the global microenvironment may result in an increased amelioration of the SR-induced cognitive deficits, either by modulating activity-dependent functions that converge on neurotransmission or improving cell type-specific processes that directly impact neural network functionality. The first report that human stem cell grafting in the irradiated rodent brain could ameliorate radiation-induced cognitive dysfunction provided some of the first evidence that locally directed interventions could have a widespread impact across large neural domains [131]. Intrahippocampal grafting of various stem cell types was found to preserve host neuronal structure, attenuate neuroinflammation, and promote improved behavioral performance across a wide range of hippocampal- and cortical-dependent tasks [132,133,134]. Interesting too was the observation that at 1, 4, and 8 months post-irradiation and grafting, only 24%, 12%, and 4% of the ~800,000 total injected cells remained, respectively [132,135,136], whereas improvements to neurocognitive functionality persisted [137]. At one month, ~11% of the grafted cells co-expressed the activity-regulated cytoskeleton-associated protein (ARC) [132], and at the later 8-month time, brief exposure to novelty stimulated a significant increase in ARC expression, but only in the irradiated hippocampi of stem cell-grafted cohorts as opposed to irradiated vehicle controls [136]. New studies have uncovered some fascinating activities of the ARC protein that suggest it forms a viral-like capsid able to protect and transport RNA across the brain to impact new and learned memory responses [138,139,140]. Should SR elicit similar changes in ARC activity or structurally related viral mimics in the brain, relatively small doses to any given neural subregion could elicit global changes in circuit connectivity through multiple mechanisms.

The foregoing also points to a potential role of secreted extracellular vesicles which contain a wealth of bioactive cargo. Extracellular vesicles (EV) or their size-restricted exosome subtypes are secreted by virtually any cell type and mediate proximal (autocrine) and distal (paracrine) signaling to modulate physiological stress responses within and across damaged tissue types [141]. Within the partially irradiated brain, translocation of secreted EV could explain compensatory responses in neurogenesis and neuronal sculpting observed at contralateral locations [142,143]. Indeed, much of the work performed with stem cell grafting in the irradiated brain can be replicated by the substitution of stem cells with stem cell-derived EV. The delivery of stem cell-derived EV into the hippocampus was found to be equally efficacious as stem cell grafting in ameliorating radiation-induced cognitive dysfunction through similar protective mechanisms on neuronal structure and abatement of the reactive microglial sensome [144]. Subsequent work identified that many of the benefits could be attributed to select miRNAs, able to cross the blood–brain barrier when administered systemically, pointing to their marked capability to traverse robust biological barriers and fuse with target cells to elicit pleiotropic effects on cellular physiology [143,145]. Although somewhat circumstantial, the foregoing does provide a plausible rationale for expectations that similar EV trafficking in the SR-exposed brain could attenuate global stress responses and impair circuit connectivity and cognitive acuity. Although such effects may be subtle under normal work cycles, unexpected emergencies, multitasking, and rapid decision-making could be unduly impacted by EV-mediated circuit changes. Again, such considerations indicate that to elicit meaningful functional change in the CNS, cell death is not a prerequisite, and the fact that cognitive outcomes in the SR-exposed rodent brain show little dependence on dose or microdosimetry only supports this tenet further.

### 3.3. Paradigm Shift 3: Diffuse as Opposed to Localized Brain Regions as a Target for Radiation Effects

Although it is convenient to classify a neurocognitive task as region-specific (e.g., hippocampus), in reality, most tasks require a highly coordinated response of multiple brain regions for successful completion. Executive functions serve as a meta-cognitive, supervisory, or controlling system [18] and require the integrated processing of information within and across neural networks. For example, at least 11 brain regions are involved in human task switching [146]. Typically, as the complexity of a task increases, so does the number of cognitive processes that are required to successfully complete that task, and thus, an increased need to efficiently coordinate the activity of the involved brain regions. Therefore, executive function performance is largely determined by multiple regions of the brain, i.e., a neural network, working in a highly coordinated manner. Moreover, behavioral performance is frequently determined by a complex interaction between neural networks that are classified as either task-negative (activity reduced during task) or task-positive (activity increased during task), which operate largely in opposition to each other.

Neuroimaging studies have identified multiple neural networks within humans, many of which are closely associated with distinct cognitive and/or psychological domains [147,148]. The major networks conceptually important for cognitive performance include a central executive network (CEN), a social brain/default mode network (DMN), and a salience/emotion processing network (SEN). The triple network model posits that the disordered coupling among the DMN, SEN, and CEN is responsible for cognitive impairment in many brain disorders [149]. Interconnectivity between networks mediates monitoring and reciprocal influences of the internal mental environment (DMN), relevant interoceptive, autonomic, and emotional information (SEN), and higher-order cognitive function and attention control (CEN) [149]. Disruption of intrinsic connectivity within and between these networks could be a core mechanism of SR-induced cognitive impairment [149]. SR (He) exposure has indeed been shown to decrease the functional connectivity between the hippocampus and perirhinal cortex [22] and also activity within hippocampal neural networks [150]. There seems little reason to suspect that this specific connection would be the only one impacted by SR.

These data have both practical and conceptual ramifications. Firstly, most of the advanced cognitive functions that impact a patient’s quality of life will be regulated by multiple regions of the brain. Given that, in many instances, good cognitive performance requires that multiple regions of the brain, i.e., a neural network, working in a highly coordinated manner, the loss of function in any node of the neural network is likely to reduce cognitive performance. Although we have primarily focused on the effects of SR in this review, as this is most pertinent to clinical radiation treatment, other space flight stressors appear to alter neural network connectivity [151]. Ongoing research has established that increased connectivity (coactivation) between certain brain regions is associated with poor cognitive performance in astronauts returning from long ISS missions, but this may be quite specific, since increased connectivity between other regions may be associated with poor cognitive performance (Schoenmaekers, NASA Human Research Program Investigators’ workshop (HRP 2023)). Similarly, there appear to be microgravity-induced changes in the DMN activity levels (Maestu, HRP 2023). Interestingly, recent work in mice exposed to the complex 33-beam GCR terrestrial simulation supports the foregoing. In situ microdialysis in the brain identified specific changes in neurotransmitter levels and response patterns within the prefrontal cortex. Interestingly, correlation and machine learning analyses revealed that such exposures differentially reorganized the connection strength and causation of dopamine and other monoamine prefrontal cortex neurotransmitter networks compared to controls (Desai-HRP2023). As animals were subjected to whole-body exposures, it is highly probable that such changes transpired throughout the brain, where SR-induced reorganization of widespread circuitry may well provide a foundation for a generalized explanation of SR-induced neurocognitive deficits. Multiple research efforts are underway to assess neural network activity changes in both rodents and humans exposed to a variety of space flight stressors.

### 3.4. Benefits to Humanity Arising from Space Radiobiology Studies

NASA’s increased interest in funding CNS space radiobiology research has yielded a wealth of unexpected surprises. As investigators at the outset of this endeavor, it is safe to say that few in the field expected such research to uncover such pronounced and persistent SR-induced deficits in cognition spanning multiple tasks and brain regions. That functional CNS change showed little dependence on dose or microdosimetry confounded stochastic models traditionally focused on DNA-damaged based cell kill that attempted to rationalize radiogenic cancer induction for SR exposures. Clearly, the functional CNS outcomes do not track models of radiogenic cancer and likely do not depend on overt cell death, and the preponderance of data supports these ideas. Much has been learned over the past decade of CNS space radiobiology studies, and the post-mitotic brain is clearly not a passive response tissue, but rather a dynamically responding organ with all cell types in structurally distinct hierarchies interacting to maintain homeostatic signaling. Sufficient data exist to suggest that SR exposure disrupts this balance and alters the balance between excitatory and inhibitory tones and does so at global levels rather than at discrete proximal circuits. Such effects are also not solely neuronal, as astroglial and microglial components reshape, survey, support, and influence all measured functional outcomes in the CNS. Challenges remain as we try to further elucidate the nuances of SR exposure on the brain, but with knowledge comes power. For now, our best strategy for forging the frontiers of space in a safe but expeditious way will be to fund targeted research efforts to understand and mitigate as much as possible the collective harmful effects of the space environment on CNS functionality.

## 4. Executive Summary

One of NASA’s stated missions is to “innovate for the benefit of humanity”. The new perspectives on how the CNS responds to SR exposure have challenged the traditional concepts that have driven the application of radiation to treat brain cancers. It may thus be germane that the radiotherapy approaches used to treat brain cancer be reevaluated in light of the paradigm shifts in CNS radiobiology that have resulted from the space radiobiology studies. 

## Figures and Tables

**Table 1:** Actual and biologically weighted threshold doses for cognitive/behavioral performance inhibition by various space radiation ions.

	^56^Fe (600 MeV/n)	^56^Fe (1 GeV/n)	^28^Si (600 MeV/n)	^20^Ne (522 MeV/n)
LET (KeV/μm)	176	151	50	28
RBE [35]	3.3	2.6	1.6	1.4
	TD/**BED** (Gy)			
Operant Response [36]	2/**6**	0.5/**1.3**	0.50/**0.80**	
Taste Aversion [36]	0.5/**1.65**	0.80/**2.08**	0.10/**0.16**	
Taste Aversion [37]	0.1/**0.33**	0.8/**2.08**		
Dopamine Release [37]	0.1/**0.33**	10/**0.26**		0.5/**0.75**
Motor Function [37]	0.1/**0.33**	10/**0.26**		1.0/**1.4**

## Data Availability

Not applicable.
